# Supplementary material for: Feasibility and Acute Toxicity of Hypo-Fractionated Radiotherapy on 0.35T MR-LINAC: The First Prospective Study in Spain
Source: Cancers (Basel). 2024 Apr 26;16(9):1685. doi: 10.3390/cancers16091685 (PMC11083553; doi:10.3390/cancers16091685)
Supplement: Supplementary file 1 [file cancers-16-01685-s001.zip › cancers-2954512-supplementary.pdf]

Supplementary Materials

**Table S1.** Hypo-fractionated MRgRT fractionation protocol scheme. Abbreviations: Fx = fractions; AD = alternate days; D = daily.

| **Localization** | **Dose** | **Target Volume** |
|---|---|---|
| **Prostate**<br>Low Risk and Intermediate favorable risk<br><br><br>Intermediate unfavorable risk and High risk<br><br>Prostate Bed<br><br><br>Reirradiation | <br>36,50Gy in 5 fx (AD)<br>60 Gy in 20 fx(D)<br><br>40Gy in 5 fx (AD)<br><br><br>32,50 in 5 fx (AD)<br><br><br>30 in 5 fx (AD) | <br>Prostate<br>Prostate (Volumen > 100 cc)<br><br>Prostate and 2cm Seminal Vesicles<br><br><br>Prostate bed |
| **Pancreas**<br><br><br><br>**Reirradiation** | 30- 50Gy in 5 fx (D)<br>36 Gy in 15 fx (D)<br><br><br><br>25- 35 Gy in 5 fx (D) | Pancreatic tumor +/- clinical elective volume ( vascular structure within 5 mm, clinical node positive,...)<br><br>Pancreatic Tumor and/ or node |
| **NSCLC**<br>**Peripheral Tumor**<br><br><br><br><br>**Central Tumor**<br>**Ultra Central Tumor** | <br>45-60 Gy in 3 fx (AD)<br>48-50 Gy in 4 fx (AD)<br>50-60 Gy in 5 fx (AD)<br>28-34 Gy in 1 fx<br><br>50- 55 Gy in 5 fx (AD)<br>60 Gy in 8 fx (AD)<br>50 Gy in 10 fx (D)<br>60 Gy in 12 fx (D) | <br>Lung Tumor |
| **Liver** | 45-60 Gy in 3 fx (AD)<br>40- 50Gy in 5 fx (AD)<br>30 Gy in 10 fx (AD) | Liver Tumor |
| **Adrenal gland** | 36Gy in 3 fx | Adrenal gland |

**Table S2.** Organs at Risk Constraints protocol for five fractions.

| Structure | Volume to dose | (Gy) | Max (Gy) | Mean (Gy) |
|---|---|---|---|---|
| **Esophagus** | ≤5cc | 32,5 | 38 | |
| **Heart** | ≤15cc | 32 | 38 | |
| **Aorta** | ≤10cc | 47 | 53 | |
| **Great Vessels** | ≤10cc | 47 | 53 | |
| **Trachea** | ≤5cc | 45 | 50 | |
| **Bronchus** | ≤5cc<br>≤0,04cc | 45<br>50 | 50 | |
| **Chestwall** | ≤5cc | 45 | 57 | |
| **Lung Total** | ≤950cc<br>≤37% | 12,5<br>13,5 | | |
| **Duodenum** | <9cc<br><3cc<br><1cc | 15<br>20<br>33 | | |
| **Liver** | <50% | 12 | | |
| **Stomach** | <50%<br><1cc | 12<br>33 | | |
| **Spinal Cord** | <1cc | 8 | | |
| **Kidney** | <200cc | 17,5 | | |
| **Bowel (Pancreas)** | <5cc | 19 | | |
| **Inferior Vena Cava** | | | 30 | |
| **Rectum** | <50%<br><50%<br><20%<br><10%<br><5% | 20<br>24<br>32<br>36<br>40 | 42 | |
| **Bowel (Prostate)** | <30cc<br><1cc | 20<br>30 | | |

| | | | | |
|---|---|---|---|---|
| **Bladder** | <40%<br><5%<br><2% | 20<br>40<br>39 | 42 | |
| **Femoral Head** | <10cc | 20 | | |
| **Penile Bulb** | <5% | 24,8 | | <16 |
| **PRV Urethra** | | | 42 | |
